# An Analysis of News Media Coverage of Complementary and Alternative Medicine

**DOI:** 10.1371/journal.pone.0002406

**Published:** 2008-06-11

**Authors:** Billie Bonevski, Amanda Wilson, David A. Henry

**Affiliations:** 1 Discipline of Clinical Pharmacology, University of Newcastle, Newcastle, New South Wales, Australia; 2 Chief Executive Officer, Institute of Clinical Evaluative Sciences, Toronto, Canada; Universidad Peruana Cayetano Heredia, Peru

## Abstract

**Background:**

To examine the accuracy and adequacy of lay media news stories about complementary and alternative medicines and therapies.

**Methodology/Principal Findings:**

A descriptive analysis of news stories about complementary and alternative medicine (CAM) in the Australian media using a national medical news monitoring website, mediadoctor.org.au. Each story was rated against 10 criteria by two individuals. Consensus scores of 222 news articles reporting therapeutic claims about complementary medicines posted on mediadoctor.org.au between 1 January 2004 and 1 September 2007 were calculated. The overall rating score for 222 CAM articles was 50% (95% CI 47% to 53%). There was a statistically significant (F = 3.68, p = 0.006) difference in cumulative mean scores according to type of therapy: biologically based practices (54%, 95% CI 50% to 58%); manipulative body based practices (46%, 95% CI 39% to 54%), whole medical systems (45%, 95% CI 32% to 58%), mind body medicine (41%, 95% CI 31% to 50%) and energy medicine (33%, 95% CI 11% to 55%). There was a statistically significant difference in cumulative mean scores (F = 3.72, p = 0.0001) according to the clinical outcome of interest with stories about cancer treatments (62%, 95% CI 54% to 70%) scoring highest and stories about treatments for children's behavioural and mental health concerns scoring lowest (31%, 95% CI 19% to 43%). Significant differences were also found in scores between media outlets.

**Conclusions/Significance:**

There is substantial variability in news reporting practices about CAM. Overall, although they may be improving, the scores remain generally low. It appears that much of the information the public receives about CAM is inaccurate or incomplete.

## Introduction

News media coverage of health issues has increased dramatically in recent years.[1] In the United States, the New York Times increased its medical articles contents by 425% between 1969 and 1988.[2] Chapman reported that in Australia too, the appetite for health news and health related television has increased.[3] Newspapers, magazines, and journal articles are often cited by the public as common sources of health information.[4], [5], [6], [7] In a National Health Council Survey in 1997, 75% of Americans reported they paid a moderate amount or a great deal of attention to medical and health news.[4] Only 5% claimed they paid no attention. It is important that news coverage of health issues is of high quality as there is substantial evidence of a link between health news reports and health behaviour.[8], [9] For example, news of Kylie Minogue's breast cancer generated a sustained 101% increase in never-screened women booking for mammograms.[9] Despite its potential to inform and educate the public about health issues and influence health behaviour, studies have found varying degrees of inaccuracies and omissions in health news stories.[10], [11], [12], [13], [14], [15] Common concerns about reporting include: unnecessary sensationalism, inadequate follow-through, failure to consider the quality of evidence, inaccurate portrayal of benefits, lack of consideration of adverse effects and costs, and the failure to obtain comments from independent informants.[10], [11], [12], [13], [14], [15]

Despite substantial growth in the use of complementary and alternative medicine (CAM) [16], [17], [18], [19], [20] very little is known about how the media reports on it. One small study, which examined the type and tone of media reporting about CAM in the UK and Germany suggested some variability in the reporting of CAM.[21] As attempts continue to generate knowledge on the efficacy and safety of CAM the media has a crucial role in communicating that information to the public.[22]

Media Doctor (www.mediadoctor.org.au) is a web-based program that monitors, rates and critiques the accuracy and completeness of health news stories in Australia. It publishes quality assessments and critiques of news articles about medical treatments. This paper aims to examine the type and quality of health news reports about CAM in the Australian media.

## Methods

A descriptive study was used to determine whether the type of variability evident in previous examinations of the quality of health news reporting exists within the field of CAM news. More specifically, we examined whether differences exist in the quality of reporting according to the type of CAM practices reported on, the clinical condition of interest and the media source reporting the CAM news. As popular awareness and use of CAM increased during the study period, we examined whether there were any improvements in news reporting about CAM over time. As our rating instrument assesses several domains we examined whether there were particular areas of strengths and weakness in reporting CAM news, according to our ten rating criteria.

### Defining CAM

Several definitions of CAM exist.[18], [23], [24], [25], [26], [27] In order to be comparable and inclusive, this paper uses the definition from the Cochrane Collaboration that CAM “includes all such practices and ideas which are outside the domain of conventional medicine in several countries and defined by its users as preventing or treating illness, or promoting health and well-being.”[26]

To categorise the different forms of CAM falling under this definition, we adopted a US-based system [24] currently used by the Australian medicines regulator the Therapeutic Goods Administration.[28] It provides the following categories:

1. Biologically-based practices (including dietary supplements, botanicals, animal-derived extracts, vitamins, minerals, fatty acids, amino acids, proteins, probiotics, whole diets and functional foods).
2. Energy medicine (including visible light, magnetism, laser beams, other electromagnetic forces, and biofields such as ki, doshas, prana, atheric energy, and mana).
3. Manipulative and body-based practices (including chiropractic manipulation, osteopathic manipulation, massage therapy, reflexology, Bowen technique, Alexander technique).
4. Mind-body medicine (relaxation, hypnosis, visual imagery, meditation, yoga, biofeedback, qi gong, cognitive behavioural therapies and spirituality)
5. Whole medical systems (including traditional Chinese medicine, ayurvedic medicine, naturopathy, homeopathy, and acupuncture).

### Selection of articles

Media Doctor collects health related articles from the major Australian news outlets (see Table 1). These media sources were chosen because they were national or state-wide in distribution, had a large circulation or audience base and represented the main forms of mainstream media in Australia; print, online and television. Articles identified through these sources are eligible for inclusion if they made therapeutic claims about new treatments, procedures and diagnostic tests. Generally, these claims were said to be based on clinical research findings.

**Table 1:** Summary of media outlets included in the current study.

Type	Media outlet
Broadsheet Newspapers	Sydney Morning Herald
	The Australian
	The Age
Tabloid Newspapers	The Daily Telegraph
	The Courier Mail
	Sunday Telegraph
	The Sun Herald
	Herald Sun
Internet News	ABC online
	ninemsn
Current Affairs Television Programs	Nine's ‘A Current Affair’
	Seven's ‘Today Tonight’

### Main outcome measure

The Media Doctor rating instrument was adapted from one previously used to assess the quality of medical news reporting in the USA [10] and is consistent with Australian Press Council recommendations.[29] It consists of ten criteria and simple dichotomous (satisfactory or not satisfactory) items. The criteria are; was the novelty of the treatment reported?, was the availability of the treatment reported?, were treatment options described?, did the story contain elements of disease mongering?, was the reporting of evidence (study methodology) included?, were benefits framed in both relative and absolute terms?, was there mention of potential harms?, was there mention of costs?, was an independent comment included?, was the story sufficiently different from the press release (where this was available)? To score as satisfactory, specified criteria had to be met. Raters were provided with detailed descriptions of how each criterion should be rated.

### Data collection

Current news articles about medical treatments including surgical, pharmaceutical, and ‘other’ treatments and diagnostic tests were identified by daily web site searches by a research assistant. Eligible articles were sent to reviewers matching article content with reviewer expertise. Two trained reviewers assessed each article. All reviewers and their credentials are listed on the website (www.mediadoctor.org.au). Generally each article was reviewed by one non-physician, health-based academic and one medical practitioner. The results of inter-reviewer agreement scores are reported elsewhere [30] and were moderate to substantial [31] (kappa scores between 0.49 and 0.74). We did not separately measure the levels of inter-rater agreement as the stories conformed to the structure of those covering non-CAM therapies and we were able to apply our rating form without modification. Consensus scores were agreed on by the two reviewers with disagreements resolved by a third party. Raters wrote short commentaries based on the criteria listed in the rating instrument. All reviews are checked by an administrator before being posted on the website. Attempts were made to locate any relevant media releases, journal articles or other supporting literature that may assist reviewers.

Total scores were posted for articles that had at least seven criterion ratings and were expressed as percentages of the theoretical maximum score. For example, if all ten criteria are scored satisfactory, the article would receive a total score of 100%. If six out of eight rated criteria were scored satisfactory and two unsatisfactory, the article would receive a total score of 75%, and so on. On the website, the total scores are translated into a star rating for general public ease of use (0 = no star, 1–20% = 1 star; 21%–40% = 2 stars; 41%–60% = 3 stars; 61–80% = 4 stars, 81–100% = 5 stars). Cumulative scores for each media outlet were posted on the website, providing ongoing feedback on their performance.

### Statistical analyses

Mean proportions and their 95% confidence intervals were calculated for each outcome of interest. The data were plotted and found to be normally distributed. Comparisons using unweighted cumulative total scores for each group were performed. Where a comparison involved more than two groups (as in the case of comparing CAM category scores, scores across media outlets, scores during the first and second time period, and clinical outcome category scores) one-way analysis of variance was used. To further examine the trend in scores over time we performed simple linear unweighted regression analysis with time to publication (in days since 31^st^ May 2004) on the horizontal axis and percentage scores for each article on the vertical axis. All statistical calculations were made using StatsDirect (version 2.3.6, Stats Direct Ltd, Sale, Cheshire, UK).

## Results

Between 1 January 2004 and 1 September 2007, 1087 articles were reviewed by Media Doctor. Of these, 557 reported ‘pharmaceutical’ treatments, 92 reported new ‘surgical’ treatments, 108 reported ‘diagnostic’ developments and 330 were classified in the ‘other’ category. Articles in the latter group were individually reviewed to determine whether they were CAM (according to the definition and categorisation described above). One hundred and six (106) of these articles were ineligible for further inclusion since they included non-CAM developments (such as dental treatments, optical treatments, preventative screening methods). Two articles were excluded because they were double entries. As a result, 222 articles (20% of the total) classified as CAM were included in the study.

Comparison of the cumulative total mean scores for the four types of articles showed that although CAM articles scored lower than other types of stories (mean total score 50%, 95% CI 47% to 53%), they were not statistically different from stories about new medicines, (53%, 95% CI 51% to 54%), surgery, (52%, 95% CI 47% to 56%); and diagnostic interventions (51%, 95% CI 47% to 55%), (F = 0.927, df = 3, p = 0.4271).

### Types of CAM treatments

The 222 articles were individually reviewed to determine their CAM category. One hundred and forty two articles (64%) reported biologically-based practices. The majority of these (101 articles) reported nutritional benefits to health (see Table 2 for examples of story headlines for each category). Eight articles (3%) reported developments in ‘energy medicine’, 28 (13%) reported news about ‘manipulative and body based practises’, 26 (12%) articles were about ‘mind-body medicine’ and 18 (8%) articles were about ‘whole medical systems’.

**Table 2:** Examples of news story headlines and cumulative rating score by CAM category for articles posted on Media Doctor Australia, January 2004 to September 2007.

CAM Category	N (%) articles	5 Typical Headlines	Rating score (95% confidence intervals)
Biological	142 (64%)	Trial looks at mushroom's effect on blood pressure.	54% (50% to 58%)
		Tomato and broccoli recipe to fight cancer.	
		The good oil on Alzheimer's.	
		Eating fish can help make brighter babies.	
		Herbal remedy eases SARS: study.	
Manipulative body-based	26 (12%)	Pumping iron halts diabetes.	46% (39% to 54%)
		Osteopathy may reduce tension headaches.	
		A new way to treat arthritis.	
		Stress training can help lower blood pressure.	
		Good news for bad backs.	
Whole medical systems	18 (8%)	Acupuncture linked to IVF success.	45% (32% to 58%)
		Chinese herbs provide period pain relief.	
		Acupuncture effective post-surgery medicine.	
		Acupuncture reduces knee pain.	
		Homeopathy ineffective, study finds.	
Mind body medicine	24 (11%)	Yoga eases period stress.	41% (31% to 50%)
		Meditation sharpens brain: scientists.	
		Brain workout slows ageing.	
		Psychotherapy aids teen diabetics: study.	
		Space technology could provide ADHD cure.	
Energy medicine	8 (4%)	New autism treatment: cruel or effective?	33% (11% to 55%).
		Microwave your flab goodbye.	
		Magnet therapy.	
		The doctor many believe can cure cancer.	
		Shock wave useful for stress fractures	

The total rating scores were compared between groups. The highest scoring category was the ‘biologically based practises’ (55%, 95% CI 50% to 58%) and the lowest scoring category was ‘energy medicine’ (33%, 95% CI 11% to 55%). The differences between categories were found to be statistically significant (F = 3.676, df = 4, p = 0.0064).

### Types of clinical outcomes

Articles were re-classified into pragmatic groupings according to the clinical outcome that the CAM treatment was claiming to modify (see Table 3). The following 11 categories were revealed: 30 articles (13%) reported the effects of CAM on cancer; 30 (13%) articles reported the effects of the CAM on cardiovascular disease and the risk factors of blood pressure and cholesterol; 27 (12%) articles reported claims about CAM improving health, general well being, prolonging life and preventing ageing; 25 (11%) articles reported about the effects of CAM on pain management, including headaches and pre-menstrual symptoms; 22 (10%) articles reported about mental health issues including Alzheimer's, dementia and depression; 15 (7%) articles reported about CAM treatments for healthy bones and joints; 13 (6%) articles were about CAM weight loss treatments; 11 (5%) articles were about CAM treatments for paediatric behavioural or mental health concerns, predominantly autism and attention deficit hyperactivity disorder (ADHD); 10 (5%) articles were about CAM for respiratory disorders, such as asthma and ‘colds’; eight articles were about diabetes treatments; and 31 (14%) articles were classified as ‘other’ which included singular stories about a wide range of conditions including acne, ‘cellulite’, blindness, insomnia, post-surgical recovery, and multiple sclerosis.

**Table 3:** Cumulative rating scores by clinical outcomes of interest in CAM articles posted on Media Doctor Australia, January 2004 to September 2007.

Clinical outcome Category	N (%) articles	Rating score (95% confidence interval)
Cancer	30 (14%)	62% (54% to 70%)
Cardiovascular disease (and risk factors blood pressure and cholesterol)	30 (14%)	59% (51% to 66%)
Bones and joints	15 (7%)	54% (43% to 65%)
Weight loss	13 (6%)	53% (37% to 70%)
Respiratory	10 (4%)	53% (39% to 68%)
General well-being/ improved health	27 (12%)	51% (40% to 61%)
Mental health	22 (10%)	49% (39% to 60%)
Diabetes	8 (3%)	48% (35% to 61%)
Pain	25 (11%)	44% (35% to 53%)
Other	31 (14%)	36% (29% to 44%)
Paediatric behavioural/mental	11 (5%)	31% (19% to 43%)

Table 3 shows the quality rating scores for each clinical outcome category. The highest rating category was cancer (62%, 95% CI 54% to 70%) and the lowest performing category was paediatric behavioural/mental health concerns (31%, 95% CI 19% to 43%). The differences between categories were found to be statistically significant (F = 3.72, df = 10, p = 0.0001).

### Differences across media sources

Differences in rating scores were compared across the four types of media outlets; broadsheet newspapers, tabloid newspapers, online news, television current affairs shows. The highest rating media source was the broadsheet newspapers (57%, 95% CI 53% to 61%), followed by online news (49%, 95% CI 43% to 54%), tabloid newspapers (45%, 95% CI 34% to 56%) and the lowest rating media source was television current affairs programs (29%, 95% CI 22% to 36%). The differences between media sources were found to be statistically significant (F = 13.657, df = 3, p = 0.0001).

### Change over time

**Figure 1:**
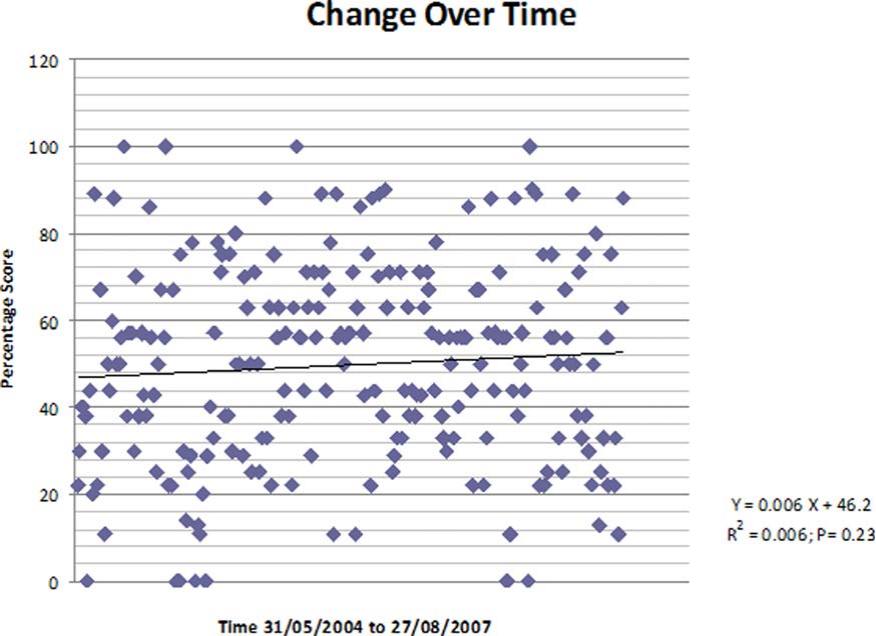
Scatterplot of change of percentage scores over time (31/05/2004 to 27/08/2007). There was an average improvement of 5.4% (95%CI-0.72, 11.6; P = 0.083), which was not statistically significant. Percentage scores were plotted over time but the slope from the regression analysis was not significantly higher than zero

To examine whether any change in rating scores has occurred over time we compared the scores for articles published before the study midpoint (13 January 2006) with those published later. There was an average improvement of 5.4% (95%CI-0.72, 11.6; P = 0.083), which was not statistically significant. Percentage scores were plotted over time but the slope from the regression analysis was not significantly higher than zero (Figure 1).

### Individual criterion scores

Individual criterion scores were examined to explore the areas where CAM articles performed well or poorly. The proportion of CAM articles rated ‘satisfactory’ for each criterion is presented in Table 4. The highest scoring criterion was absence of features of ‘disease mongering’, which was rated satisfactory in 85% of CAM articles and the lowest scoring criterion was ‘costs of therapy’, which rated satisfactory in only 15% of CAM articles.

**Table 4:** Percentage rated satisfactory for each of ten rating criteria for CAM articles posted on Media Doctor Australia, January 2004 to September 2007.

Criterion	% (and n)* rated satisfactory
Is there is evidence of ‘disease mongering’?	85% (222)
Is the treatment genuinely new?	82% (220)
Does the article rely heavily on a media release for content?	79% (57)
Does the article report the availability of the treatment in Australia?	68% (188)
Doe the article report the type of evidence supporting the treatment?	42% (222)
Are alternative treatment options mentioned?	42% (187)
How are the benefits of the treatment framed (in relative and absolute terms)?	39% (222)
Is an independent source of information or comment included?	33% (222)
Are harms of the treatment mentioned?	29% (200)
Are costs of the treatment mentioned?	15% (148)

* The denominators vary as it was not always possible to rate each criterion with the information provided in an article (receiving a not applicable score). Denominators are given in parenthesis.

## Discussion

The results show that when news stories about CAM are rated according to the extent that they meet ten widely accepted criteria, scores are variable and generally low. Scores varied according to the type of CAM therapy reported on, the clinical outcome of interest and the media source reporting the story. When reporting about CAM, it appears the media are particularly inconsistent at reporting about the costs and potential harms and benefits. The highest ratings were seen for stories about biologically based CAM treatments and treatments for cancer. The lowest ratings were associated with stories about treatments for behavioural disorders in children. The results showed that there was a small increase in ratings between 2004 and 2007, but this change of around 5% did not reach statistical significance. Overall, the data show that the public are being poorly served by some media outlets, particularly current affairs television programs.

It is important to highlight that this study is not providing any comment on the efficacy or safety of CAM or on the quality of CAM research, but rather on the media portrayal of CAM. The aim of this study was to examine the quality, accuracy and comprehensiveness of media reporting of CAM. In that regard the study provides a number of potentially important conclusions.

### How well is CAM news being reported?

The results show that the biological group of CAM therapies appear to be viewed by the media in a similar way to conventional medical treatments and reporting scores were similar (54% and 52% respectively). Other forms of CAM, particularly the energy medicine and mind body medicine forms were poorly reported. This may be due to a lack of evidence or an uncritical view on the part of the media. The latter groups contained stories about CAM therapies such as meditation, magnet therapy, yoga, electric shocks, shock waves and visualisation. It may be difficult for journalists to access adequate and accurate information about these therapies.

The largest number of CAM stories covered treatments for cancer and heart disease. These stories were better reported than others. It is disconcerting that stories about CAM therapies for mental health, diabetes, pain, and children's behavioural and mental health concerns scored well below average. To help illustrate these differences, Box S1 shows an example of a high scoring article, and a low scoring example. It is difficult to understand why there would be differences in reporting standards for different health concerns. The evidence here suggests that claims of the success of CAM in treating some conditions are being inadequately scrutinised. There appears to be the need for universal standards which should apply to all health news reporting regardless of what they are reporting about and where it is published.

Examination of individual criterion scores showed that six of the ten criteria scored less than 50% satisfactory (see Table 4). Similar observations have been made in overseas studies of health news reporting about new drugs [10], [14] and mammography screening.[32] Most stories failed to mention the costs and potential harms of the CAM treatment. These results are concerning, given the limited amount of information about the safety of many CAMs, [33] and the potential for some to interact with conventional medicines.[34] Almost two thirds of stories failed to gain a comment from an independent source or expert. Information from independent sources has the potential to offer balance in a story. Most articles that quantified the benefits of CAM framed them in relative terms which can give an overly optimistic impression of efficacy. Decisions about medical treatments are often made by balancing harms and benefits. Research has shown that most people, including clinicians, choose interventions whose benefits are framed in relative rather than absolute terms.[35], [36]

The variation in scores across media outlets is consistent with previous results about health news reporting in general.[37] In 2005, Media Doctor reported the results of the analysis of its first 104 health news articles.[30] In that study, the print media significantly outperformed online news services (overall mean scores of 56.1% and 40.1%, respectively). The earlier study was limited by the inclusion of only five media outlets (three national newspapers and two online news services). The current study has a number of advantages including larger sample size, greater specificity (examining CAM stories only), and coverage of a wider media base.

Overall, we found that broadsheet newspapers scored higher than current affairs programs. These results mirror previous research which found that “hard” news reports are generally more accurate than feature stories [38] and that print media are more accurate than television.[39] Regardless of the type of media, each of these outlets is responsible for the mass communication of health information and it would seem the challenge is to develop ways to lower the variability with which health news is reported.

### Can health news reporting about CAM improve?

Media doctor provides a minimal, passive form of feedback to interested journalists via the provision of broad media outlet scoring trends over time on the website. We found no convincing evidence of improvement in the reporting of CAM during the study period, but a controlled parallel intervention, or formal time series analysis of a more active feedback program would be needed in order to draw any confident conclusions about the potential for improvement. However there is indirect evidence that the situation could be improved. Large differences in scores between media outlets indicate that some journalists are capable of writing excellent stories about CAM. Of the 222 articles analysed in this study, four achieved scores of 100%, suggesting that it is possible to meet all the criteria. These articles included discussions about the novelty and availability of the new treatment, its costs and potential harms, evidence about its effectiveness and the appropriate framing of data on benefits. They included comments from individuals with no conflict of interest, avoided disease mongering and did not rely heavily on the press release for the content of the story. A further 19 articles achieved scores between 80–99% suggesting that it is possible to meet most of the criteria.

Some of the barriers often cited for the shortcomings in reporting include editorial pressures to produce short stories quickly [13], lack of health news specific training [40], inadequate press releases from the scientific community [41], a focus on the controversial and exciting story [42], and a lack of high level evidence for CAM in general.[43] Feedback and education for the health media may address some of the reported barriers to optimal health reporting. There is a need to change the methods of promoting research findings within the scientific community, and a need to improve training for health journalists.[44] It is clear that feedback interventions need to be more active, tailored, intensive forms of feedback and education to produce more pronounced changes.

### Limitations of the study

There are a number of limitations to the generalisability of our findings. Firstly, as a result of categorising the data, some comparison groups involved low numbers of news articles. It should be noted that this study is the largest of its type. Secondly, although attempts were made during the study period to collect all eligible news stories, some may have eluded capture due to resource limitations. However, the effects of incomplete sampling were random and we are confident that the study provides a broad and representative sample of CAM stories in the Australian media. General reporting standards generally appear to be similar in other countries.[10], [14] Thirdly, the rating instrument used for CAM was one developed for use with stories about more conventional medical interventions. Although evaluated, it is possible the rating instrument may have missed some important CAM-specific concerns or questions.

### Conclusions

This study shows that there is substantial variability in the news reporting about complementary and alternative medicines and therapies. Overall, scores were generally low and the small improvement noted during the study period was not statistically significant. Currently, it appears that much of the information the public receives about CAM is inaccurate or incomplete. The development of strategies aimed at improving health news reporting deserves more focused attention from both the media and researchers.

## Supporting Information

Box S1 Examples of high and low scoring articles in the fields of cancer treatments and child health treatments.
(0.04 MB DOC)

## References

[1] Lupton D (1995). Medical and health stories on the Sydney Morning Herald's front page.. Aust J Public Health.

[2] Wilkes MS (1997). The public dissemination of medical research: problems and solutions.. J Health Commun.

[3] Chapman S, Dominello A (2001). A strategy for increasing news media coverage of tobacco and health in Australia.. Health Promot Int.

[4] (1997). Roper Starch Worldwide Inc: Americans talk about science and medical news: The National Health Council Report. New York..

[5] Rutten LJ, Arora NK, Bakos AD, Aziz N, Rowland J (2005). Information needs and sources of information among cancer patients: a systematic review of research (1980–2003).. Patient Educ Couns.

[6] Hann DM, Baker F, Roberts CS, Witt C, McDonald J (2005). Use of complementary therapies among breast and prostate cancer patients during treatment: a multisite study.. Integr Cancer Ther.

[7] Dolan G, Iredale R, Williams R, Ameen J (2004). Consumer use of the internet for health information: a survey of primary care patients care patients.. Int J Consumer Studies.

[8] Haas JS, Kaplan CP, Gerstenberger EP, Kerlikowske K (2004). Changes in the use of postmenopausal hormone therapy after the publication of clinical trial results.. Ann Intern Med.

[9] Chapman S, McLeod K, Wakefield M, Holding S (2005). Impact of news of celebrity illness on breast cancer screening: Kylie Minogue's breast cancer diagnosis.. Med J Aust.

[10] Moynihan R, Bero L, Ross-Degnan D, Henry D, Lee K (2000). Coverage by the news media of the benefits and risks of medications.. N Engl J Med.

[11] Chapman S, Lupton D (1994). Freaks, moral tales and medical marvels: health and medical stories on Australian television.. Media Information Australia.

[12] Shuchman M, Wilkes MS (1997). Medical scientists and health news reporting: a case of miscommunication.. Ann Intern Med.

[13] Schwartz LM, Woloshin S (2004). The media matter: a call for straightforward medical reporting.. Ann Intern Med.

[14] Cassels A, Hughes MA, Cole C, Mintzes B, Lexchin J (2003). Drugs in the news: an analysis of Canadian newspaper coverage of new prescription drugs.. Cmaj.

[15] Woloshin S, Schwartz LM (2006). Media reporting on research presented at scientific meetings: more caution needed.. Med J Aust.

[16] MacLennan AH, Wilson DH, Taylor AW (2002). The escalating cost and prevalence of alternative medicine.. Prev Med.

[17] Thomas KJ, Nicholl JP, Coleman P (2001). Use and expenditure on complementary medicine in England: a population based survey.. Complement Ther Med.

[18] Eisenberg DM, Davis RB, Ettner SL, Appel S, Wilkey S (1998). Trends in alternative medicine use in the United States, 1990-1997: results of a follow-up national survey.. JAMA.

[19] Cohen MM, Penman S, Pirotta M, Da Costa C (2005). The integration of complementary therapies in Australian general practice: results of a national survey.. J Altern Complement Med.

[20] Australian Medical Association (2002). Complementary Medicine Position Statement. Australian Medical Association..

[21] Ernst E, Weihmayr T (2000). UK and German media differ over complementary medicine.. BMJ.

[22] Vickers A (2000). Recent advances: complementary medicine.. Bmj.

[23] Caspi O, Sechrest L, Pitluk HC, Marshall CL, Bell IR (2003). On the definition of complementary, alternative, and integrative medicine: societal mega-stereotypes vs. the patients' perspectives.. Altern Ther Health Med.

[24] National Centre for Complementary and Alternative Medicine (2002). What is complementary and alternative medicine (CAM)?.

[25] Commonwealth of Australia (2003). Complementary Medicines in the Australian Health System. Expert Committee on Complementary Medicines in the Health System. Report to the Parliamentary Secretary to the Minister of Health and Ageing. Commonwealth of Australia..

[26] Berman BM (1997). The Cochrane Collaboration and evidence-based complementary medicine.. J Altern Complement Med.

[27] House of Lords Select Committee on Science and Technology (2000). Session 1999–2000, 6th Report, Complementary and alternative Medicine..

[28] Commonwealth of Australia (2003). Australian Medical Devices Guidelines, The Use of Medical Devices in Alternative Therapies. Therapeutic Goods Administration..

[29] Australian Press Council (2001). Reporting guidelines.. General press release No 245 (April 2001).

[30] Smith DE, Wilson AJ, Henry DA (2005). Monitoring the quality of medical news reporting: early experience with media doctor.. Med J Aust.

[31] McGinn T, Wyer PC, Newman TB, Keitz S, Leipzig R (2004). Tips for learners of evidence-based medicine: 3. Measures of observer variability (kappa statistic).. CMAJ.

[32] Wells J, Marshall P, Crawley B, Dickersin K (2001). Newspaper reporting of screening mammography.. Ann Intern Med.

[33] Ernst E, Leith G, Jonas W, Walach H (2002). Investigating the safety of complementary medicine.. Clinical Research in Complementary Therapies: Principles, Problems and Solutions.

[34] Woodward KN (2005). The potential impact of the use of homeopathic and herbal remedies on monitoring the safety of prescription products.. Hum Exp Toxicol.

[35] Hux JE, Naylor CD (1995). Communicating the benefits of chronic preventive therapy: does the format of efficacy data determine patients' acceptance of treatment?. Med Decis Making.

[36] Bucher HC, Weinbacher M, Gyr K (1994). Influence of method of reporting study results on decision of physicians to prescribe drugs to lower cholesterol concentration.. BMJ.

[37] Bubela TM, Caulfield TA (2004). Do the print media “hype” genetic research? A comparison of newspaper stories and peer-reviewed research papers.. CMAJ.

[38] Henderson L, Kitzinger J (1999). The human drama of genetics: “hard” and “soft” media representations of inherited breast cancer.. Social Health & Illness.

[39] Condit CM, Ofulue N, Sheedy KM (1998). Determinism and mass-media portrayals of genetics.. Am J Hum Genet.

[40] Voss M (2002). Checking the pulse: Midwestern reporters' opinions on their ability to report health care news.. Am J Public Health.

[41] Woloshin S, Schwartz LM (2002). Press releases: translating research into news.. JAMA.

[42] Johnson T (1998). Shattuck lecture–medicine and the media.. N Engl J Med.

[43] Fontanarosa PB, Lundberg GD (1998). Alternative medicine meets science.. JAMA.

[44] Ransohoff DF, Ransohoff RM (2001). Sensationalism in the media: when scientists and journalists may be complicit collaborators.. Eff Clin Pract.

