# Supplementary material for: An Analysis of News Media Coverage of Complementary and Alternative Medicine
Source: PLoS One. 2008 Jun 11;3(6):e2406. doi: 10.1371/journal.pone.0002406 (PMC2405931; doi:10.1371/journal.pone.0002406)
Supplement: Box S1 — Examples of high and low scoring articles in the fields of cancer treatments and child health treatments. (0.04 MB DOC) [file pone.0002406.s001.doc]

**Box 1. Examples of high and low scoring articles in the fields of cancer treatments and child health treatments.**

| "Study links red meat to breast cancerA Harvard Medical School study has discovered a link between red meat consumption and breast cancer.”Total score 88 **Novelty of Treatment** Satisfactory  **Availability of Treatment** Satisfactory  **Treatment Options** Not Applicable  **Disease Mongering** Satisfactory  **Evidence** Satisfactory  **Quantification of Benefits of Treatment** Not Satisfactory  **Harms of Treatment** Satisfactory  **Costs of Treatment** Not Applicable  **Sources of Information** Satisfactory  **Relies on Press Release** Satisfactory  **Reviewers comments:**  This story contains a lot more independent comment than that from AAP/ Reuters, which appeared in a number of media outlets. As a result it is a better story. The main problem is that the informants are trying to provide practical advice (some of it possibly conflicted by commercial interests) when the research itself only shows an association, not causation. In our view more time should have been spent commenting on the limitations of the study design and the need for replication of the results. |
| --- |
| "New autism treatment: cruel or effective? It may seem like cruel and unusual punishment, but intense electric shocks can help parents control children made violent and aggressive by autism, says one expert.”  **Total score: 11**  **Novelty of Treatment** Not Satisfactory  **Availability of Treatment** Not Satisfactory  **Treatment Options** Not Satisfactory  **Disease Mongering** Satisfactory  **Evidence** Not Satisfactory  **Quantification of Benefits of Treatment** Not Satisfactory  **Harms of Treatment** Not Satisfactory  **Costs of Treatment** Not Satisfactory  **Sources of Information** Not Satisfactory  **Relies on Press Release** Not Applicable  **Reviewers comments:**  This is a concerning piece of health reporting. There is no evidence that this treatment, consisting of electric shocks to children, is effective at treating autism. There is a good amount of evidence showing that it is painful. I am not sure how this treatment is allowed to be used since if a parent chose to do this to their child, child welfare agencies would probably rightly step in and stop it. There's no description of autism and what treatments are available. We only have the treatment creators word for it when he says there are no side effects. I would like to see some more independent research on the psychological and physical effects of electric shocks on children who probably don't understand what is happening to them. Arguing that electric shocks are better than banging heads on walls is insufficient; I would like to see evidence guaranteeing that the treatment works AND has no side effects before inflicting on children. |
